# Fast Motion Deblurring Using Sensor-Aided Motion Trajectory Estimation

**DOI:** 10.1155/2014/649272

**Published:** 2014-11-04

**Authors:** Eunsung Lee, Eunjung Chae, Hejin Cheong, Joonki Paik

**Affiliations:** Department of Image, Chung-Ang University, Seoul 156-756, Republic of Korea

## Abstract

This paper presents an image deblurring algorithm to remove motion blur using analysis of motion trajectories and local statistics based on inertial sensors. The proposed method estimates a point-spread-function (PSF) of motion blur by accumulating reweighted projections of the trajectory. A motion blurred image is then adaptively restored using the estimated PSF and spatially varying activity map to reduce both restoration artifacts and noise amplification. Experimental results demonstrate that the proposed method outperforms existing PSF estimation-based motion deconvolution methods in the sense of both objective and subjective performance measures. The proposed algorithm can be employed in various imaging devices because of its efficient implementation without an iterative computational structure.

## 1. Introduction

Restoration of motion blurred images is a fundamental problem of image processing especially under a poor illumination condition, where a long exposure creates unwanted motion blur. A number of blind image deconvolution methods have been proposed to remove motion blur. In this context practical blind image deconvolution can be categorized into three main varieties: single image-based, multiple image-based, and hardware-aided approaches.

Single image-based blind deconvolution estimates the blur kernel in the form of a point-spread-function (PSF) based on a simple parametric model using a single input image [1, 2]. However, a simple parametric curve cannot successfully represent the motion PSF made by various types of real camera motions. Fergus et al. proposed a general motion PSF estimation method which uses a sophisticated variational Bayesian method based on the natural image prior [3], which was followed up by related research in [4–8]. Although these methods provide a generalized camera motion model, a manual process of tuning parameters and high computational load are their disadvantages.

The multiple image-based blind deconvolution removes motion blur by appropriately combining long- and short-exposure images under the assumption that both images are captured from the same scene at the same time [9–11]. If the simultaneous acquisition assumption does not hold, the multiple image-based approach fails to remove motion blur.

The hardware-aided approach uses additional optical devices or electronic systems to overcome the limitations of the multiple image-based approach [12–16]. In spite of acquiring more accurate, robust data to estimate the motion PSF, the hardware-aided method needs a complicated optical system such as a coded-exposure or an embedded inertial sensor. An efficient implementation method of a built-in inertial sensor was introduced by Šindelář and Šroubek for mobile imaging devices [16]. But the performance of motion deblurring is not good enough because of the sensor noise and the use of a simple restoration filter.

For fast motion deblurring, both PSF estimation and the corresponding image restoration should be fast and accurate. In this paper, an adaptive image deblurring method is presented by generating the motion trajectory in the probabilistic manner and performing image restoration based on the local statistics to solve common issues in the deconvolution process. The contribution of the proposed research is twofold: (i) a novel motion PSF estimation method is proposed by minimizing the motion trajectory error based on* a priori* probability distribution, and (ii) a noniterative adaptive image restoration algorithm is proposed based on the local statistics of image to reduce ringing artifacts and noise amplification. The proposed method can quickly estimate the motion PSF using an inertial sensor and* a priori* probability distribution. The proposed adaptive image restoration algorithm minimizes restoration artifacts resulting from inaccurately estimated PSF. Both theoretical justification based on the image degradation model incorporating the projected camera motion and experimental results demonstrate that the proposed method outperforms existing state-of-the-art deconvolution methods.

## 2. Image Degradation Model Using Projected Camera Motion

**Figure 1:**
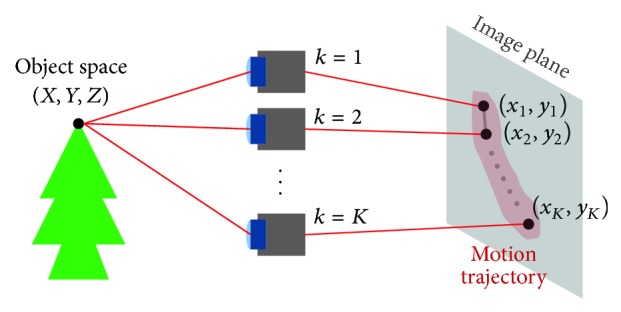
Motion trajectory generation process.

Long-exposure photography is generally degraded by motion blur. If an inertial sensor samples *K* different poses of the shaky camera during the exposure period, an object point (*X*, *Y*, *Z*) in the three-dimensional (3D) object space is projected onto *K* different positions (*x*_*k*_, *y*_*k*_),  *k* = 1,…, *K*, in the two-dimensional (2D) image plane as shown in Figure 1. More specifically, the image point is related with the object point using the homogeneous vectors as

(1) $$\begin{array}{c} {\left[x_{k},y_{k},1\right]}^{T}=\Pi_{k}{\left[X,Y,Z,1\right]}^{T}, \end{array}$$

where Π_*k*_ represents the projection matrix of the *k*th camera pose. If the motion trajectory is generated in the space-invariant manner, *K* points in the image plane generate the point-spread-function (PSF) of the corresponding motion blur as

(2) $$\begin{array}{c} h\left(m,n\right)=\frac{1}{K}\overset{K}{\underset{k=1}{\sum }}\delta \left(m-x_{k},n-y_{k}\right). \end{array}$$

Given the space-invariant PSF, the image degradation model of motion blur is given in the vector-matrix form as

(3) $$\begin{array}{c} g=Hf+\eta , \end{array}$$

where *g* represents the motion blurred image, *H* is the degradation matrix, *f* is the ideal image without motion blur, and *η* is additive noise. Assuming that the image size is *N* × *N*, all *g*, *f*, and *η* are expressed by *N* ^2^ × 1 lexicographically ordered vectors, and *H* is an *N* ^2^ × *N* ^2^ block circulant matrix defined by the PSF. In this work, we analyze the motion trajectory using inertial sensors and then compute the projection matrices. To estimate the motion PSF, each of scene points is projected into the image plane according to the projection matrices.

## 3. PSF Estimation Using Camera Motion Tracking

### 3.1. PSF Estimation of Motion Blur Based on the Projected Trajectory

**Figure 2:**
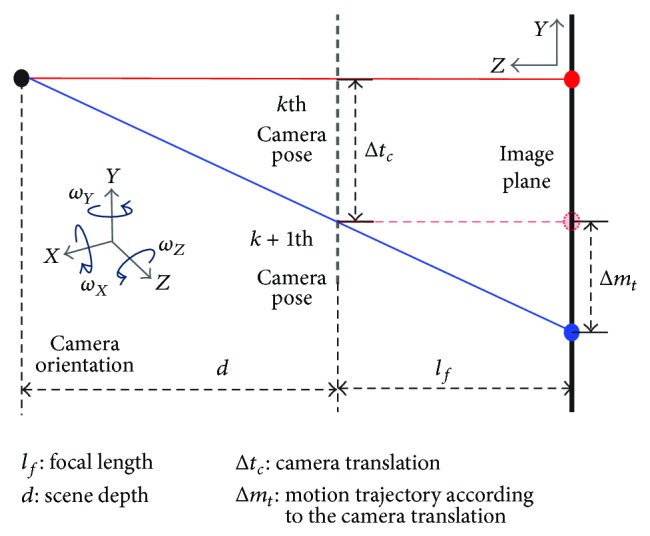
Motion trajectory according to the camera translation.

In estimating the size and shape of a motion PSF, only the relative position of the camera is needed because the PSF is the sum of reflected intensities from the first position to the last one of the camera motion as described in (2). Each camera position is projected onto the image plane and can be expressed using a planar homography as

(4) $$\begin{array}{c} {\left(x_{k},y_{k},1\right)}^{T}=\left[C\left(R_{k}+\frac{1}{d}t_{k}{n_{v}}^{T}\right)C^{-1}\right]{\left(x_{0},y_{0},1\right)}^{T}, \end{array}$$

where **C** represents the camera intrinsic matrix, **R** is the rotation matrix, *d* is the scene depth, **t** is the translation vector, and **n**_*v*_ is the normal vector to the image plane. The relationship between the motion trajectory and camera translation is shown in Figure 2, where the motion trajectory Δ*m*_*t*_ in the image plane is computed as

(5) $$\begin{array}{c} \Delta m_{t}=\frac{l_{f}}{d}\Delta t_{c}, \end{array}$$

where *l*_*f*_ and Δ*t*_*c*_, respectively, denote a focal length and translation of the camera. If the scene depth is assumed to be much larger than the focal length, Δ*m*_*t*_ can be neglected. For this reason, the camera translation does not affect the motion PSF under the large scene depth, and (4) is simplified as

(6) $$\begin{array}{c} {\left(x_{k},y_{k},1\right)}^{T}=\left[CR_{i}C^{-1}\right]{\left(x_{0},y_{0},1\right)}^{T}. \end{array}$$

The camera coordinate is assumed to be aligned to the world coordinate whose origin lies on the optical axis of the camera. In this case, camera matrix **C** is determined by the focal length *l*_*f*_ as

(7) $$\begin{array}{c} C=\left[\begin{array}{ccc} l_{f} & 0 & 0\\ 0 & l_{f} & 0\\ 0 & 0 & 1 \end{array}\right]. \end{array}$$

Using the small-angle approximation [17] and space-invariant motion blur, the rotation matrix is computed as

(8) $$\begin{array}{c} R=\left[\begin{array}{ccc} 1 & 0 & \omega_{i}^{y}\\ 0 & 1 & -\omega_{i}^{x}\\ -\omega_{i}^{y} & \omega_{i}^{x} & 1 \end{array}\right], \end{array}$$

where *ω*_*i*_^*x*^ and *ω*_*i*_^*y*^ represent the *i*th angular velocities around *x* and *y* axes, respectively. Since *l*_*f*_tan(*ω*) ≈ *l*_*f*_ *ω* for a very small *ω*, the projection matrix in (6) can be expressed as

(9) $$\begin{array}{c} {\left(x_{k},y_{k},1\right)}^{T}=\left[\begin{array}{ccc} 1 & 0 & l_{f}\omega_{i}^{y}\\ 0 & 1 & l_{f}\omega_{i}^{x}\\ 0 & 0 & 1 \end{array}\right]{\left(x_{0},y_{0},1\right)}^{T}. \end{array}$$

**Figure 3:**
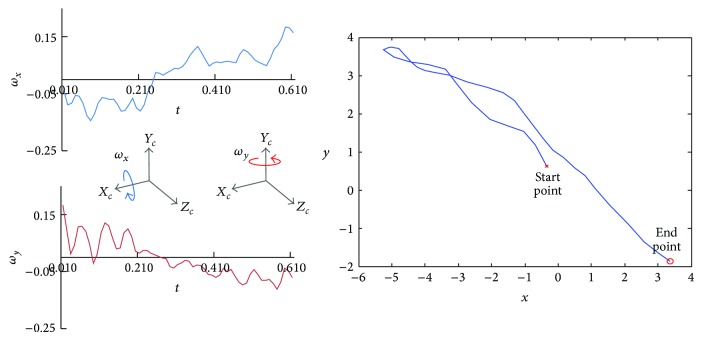
Gyro data and the projected trajectory.

**Figure 4:**
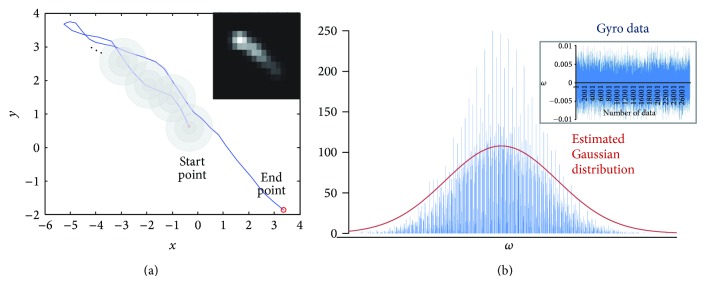
(a) The motion PSF estimation using the reweighted trajectory and (b) estimation of Gaussian distribution using the gyro data of a fixed camera shown in the rectangular box.

In this work, we use gyro data to estimate angular velocities according to the camera motion as shown in Figure 3 and compute correspondingly the projected positions in the image plane. Under the ideal condition, the projected trajectory is equal to the PSF of the camera motion. However, the gyro data are noisy under real circumstances. More specifically, noisy gyro data results in erroneous matching between the projected position in the image plane and the real PSF sample. For robust estimation of PSF using noisy gyro data, we assume that a point on the projected trajectory has Gaussian distribution, and as a result the projected trajectory consists of sum of Gaussian distributions as

(10) $$\begin{array}{c} h\left(m,n\right)=\frac{1}{K_{G}}\overset{K}{\underset{k=1}{\sum }}G\left(m-x_{k},n-y_{k}\right), \end{array}$$

where *G* represents a two-dimensional Gaussian distribution and *K*_*G*_ is the normalization constant. As a result, the PSF of camera motion becomes the accumulation of the reweighted trajectory using Gaussian distribution as shown in Figure 4(a). Gaussian distribution is estimated by analyzing the gyro data of a fixed camera as shown in Figure 4(b).

In this paper, we use “Sensor Data Logger” proposed in [18] to acquire gyro data which are synchronized with blurred frames. The gyro data and the corresponding blurred frame are time stamped, and both opening and closing times of the shutters are recorded to analyze the delay. In this paper, the unknown delay is experimentally determined for the test device.

### 3.2. Spatially Adaptive Image Restoration Using Local Statistics

Given the estimated PSF, the motion deconvolution becomes a simple image restoration problem. In recent years, many image restoration methods have been proposed to remove various types of image degradation factors. Since image restoration is an ill-posed problem, the regularized solution often requires computationally expensive iterative optimization. To remove motion blur without undesired artifacts, a novel image restoration method is presented using local statistics of the image by minimizing the energy function defined as

(11) Ef=12Hf−g22+λ12∑i=12Dif22 +Wm∘λ22∑i=12Dif−DiCg22,

where ‖·‖ denotes the Euclidean norm, “∘” is the element-wise multiplication operator, *W*_*m*_ is the spatially varying activity map, *C* is a highpass filter, *λ*_1_ and *λ*_2_, respectively, are the horizontal and vertical regularization parameters, and *D*_1_ and *D*_2_, respectively, are the horizontal and vertical derivative operators. If the estimated *f* has artifacts such as ringing or noise amplification, *D*_*i*_ *f* has sharp transitions, and as a result ‖*D*_*i*_ *f* − *D*_*i*_ *Cg*‖ becomes large.

The solution of the minimization problem is obtained by solving the equation that makes the derivative of (11) become zero, such as

(12) $$\begin{array}{c} Tf-b=0, \end{array}$$

where

(13) T=HTH+λ1∑i=12DiTDi +λ2Wm∘∑i=12DiTDi,

(14) $$\begin{array}{c} b=\left(H^{T}+\lambda_{2}W_{m}\circ \left(\overset{2}{\underset{i=1}{\sum }}D_{i}^{T}D_{i}\right)C\right)g. \end{array}$$

Since ringing artifacts appear near edges and boundaries, a spatially adaptive activity map is used to reduce the ringing artifacts while preserving edges. The proposed activity map is computed as [19]

(15) $$\begin{array}{c} W_{m}\left(x,y\right)=\frac{1}{p_{t}\sigma_{l}^{2}\left(x,y\right)+1}, \end{array}$$

where *σ*_*l*_^2^ represents the local variance in the neighborhood of (*x*, *y*) in the input image and *p*_*t*_ is a tuning parameter that makes the activity map distribute as evenly as possible in [0,1]. In this work, *p*_*t*_ = 1500 was used for the empirically best result with 5 × 5 blocks for the local variance.

Since matrix *T* is block-circulant for a space-invariant motion PSF as shown in (13), the linear equation in (12) can be solved using the two-dimensional (2D) discrete Fourier transform (DFT). Let $\tilde{f}$, $\tilde{g}$, $\tilde{h}$, $\tilde{d}$, ${\tilde{w}}_{m}$, and $\tilde{c}$ be the DFTs of the estimated image, observed image, PSF, derivative filters, activity map, and highpass filter, respectively; then the solution of the restoration problem is given as

(16) f~(k,l) =h~∗k,l+λ2w~mk,l∑i=12d~ik,l2c~k,l‍h~k,l2+λ1∑i=12d~ik,l2‍+λ2w~mk,l∑i=12d~ik,l2‍.

The finally restored image $\hat{f}$ is obtained by the inverse DFT of $\tilde{f}$.

## 4. Experimental Results

The proposed motion deblurring method is tested using indoor and outdoor images of size 1280 × 960 acquired by a smartphone with Android OS and a 2.26 GHz application processor (AP). The performance of restoration is evaluated using the no-reference image quality assessment method proposed in [20] and the CPU processing time in a personal computer equipped with 3.40 GHz CPU and 16 GB RAM. The proposed method is also compared with two types of state-of-the-art methods including the single image-based [3, 5, 7] and hardware-aided approaches [16]. The gyro data in the smartphone are measured during the exposure time.

**Figure 5:**
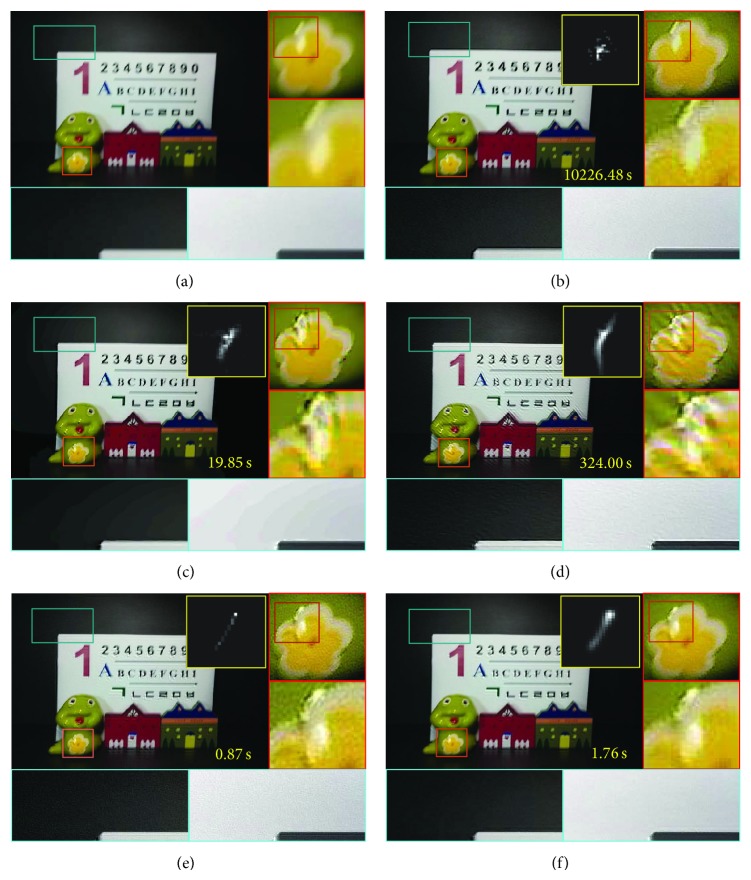
Comparison of different image restoration methods: (a) input motion-blurred image, (b) Fergus's method [3], (c) Cho's method [7], (d) Shan's method [5], (e) Šindelář's method [16], and (f) the proposed method.

Figure 5 shows restored results using different blind deconvolution methods. Although Cho's method [7] can remove motion without ringing artifact, it has unnatural discontinuities and intensity saturation due to the bilateral filtering as shown in Figure 5(c). On the other hand, the proposed method can successfully remove the motion blur without unnatural discontinuities and preserve edge regions. The proposed method also outperforms Šindelář's method [16] due to Gaussian distribution-based trajectory estimation and adaptive image restoration as shown in Figures 5(e) and 5(f).

**Figure 6:**
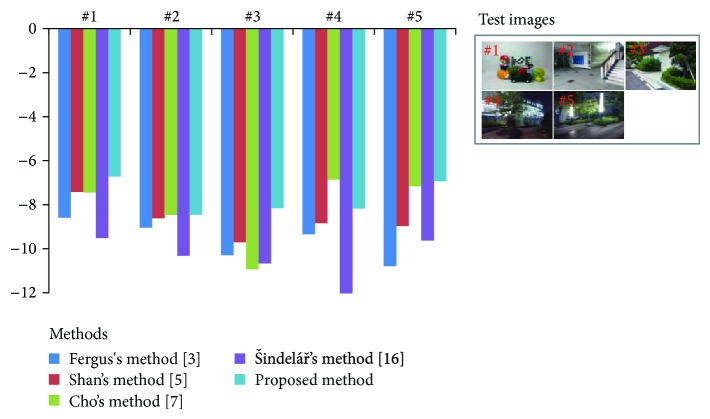
Comparison of different methods using Liu's method [20].

Figure 6 shows results of quantitative analysis using five 1280 × 960 test images. Since the proposed method needs the gyro data, quantitative analysis uses a no-reference metric of Liu's method that estimates the quality of motion deblurring. A large value of Liu's measure implies the high-quality. The result of the proposed method is comparable to or better than other deblurring methods as shown in Figure 6.

Table 1 shows processing times of five different methods. The proposed method is the fastest except Šindelář's method that uses simple Wiener filter. However the proposed method produces 26% higher deblurring measure than Šindelář's method at the cost of approximately twice longer processing time. Generally, accurate camera calibration and synchronization of gyro data are not easy tasks. The proposed motion deblurring method provides the solution for both accurate PSF estimation and image restoration using gyro data.

## 5. Conclusion

We have presented a novel motion trajectory estimation method using an embedded inertial sensor and a spatially adaptive image restoration algorithm for motion deblurring. For robust estimation of the motion PSF in the presence of sensor noise, the proposed method accumulated point-spread-functions (PSFs) of all camera positions using the projected trajectory based on Gaussian distribution. Based on the estimated motion PSF, the proposed motion deblurring algorithm can restore the image without undesired artifacts and noise amplification. The computational structure of the proposed algorithm does not need iterative minimization but uses the discrete Fourier transform domain filtering including local statistics-based spatially adaptive filtering. Since the proposed method estimates the motion trajectory using the embedded gyro sensor and performs restoration in the Fourier domain, it is much faster than existing state-of-the-art methods. Experimental results proved the performance of the proposed method in the sense of both image quality and the processing time. The future work will include a motion trajectory estimation using sensors according to scene depth for further improving the restoration performance.

## Figures and Tables

**Table 1:** Comparison of processing times of five different restoration algorithms (sec.).

Methods	Image 1	Image 2	Image 3	Image 4	Image 5
Fergus et al. [3]	10023.9	7437.4	9624.3	13353.0	8282.1
Shan et al. [5]	261.0	275.0	287.0	289.0	298.0
Cho and Lee [7]	20.1	20.4	20.7	20.3	20.4
Šindelář and Šroubek [16]	0.7	0.7	0.8	0.8	0.8
Proposed method	1.9	1.7	1.7	1.7	1.7
